# Temporal phase coverage and patient cohort size in deep learning-based cardiac cine computed tomography angiography segmentation

**DOI:** 10.3389/frai.2026.1914255

**Published:** 2026-10-05

**Authors:** Andrew Heller, Li-Yueh Hsu, An-Yu Sun, Matthew Jacobs, Lin-Ching Chang, Marcus Y. Chen

**Affiliations:** 1 National Heart, Lung, and Blood Institute, National Institutes of Health, Bethesda, MD, United States; 2 Department of Computer Science, Catholic University of America, Washington, DC, United States

**Keywords:** 3D U-Net, annotation efficiency, cardiac computed tomography angiography, cardiac segmentation, cine computed tomography angiography, deep learning, patient cohort size, temporal phase coverage

## Abstract

**Background:**

Annotating all phases of cine computed tomography angiography (CTA) is burdensome. We evaluated how temporal phase coverage and patient cohort size affect deep learning-based cardiac segmentation.

**Methods:**

Twelve 3D U-Net models were trained using a 110-patient single-phase CTA dataset or a 20-patient, 20-phase cine CTA dataset. Phase-coverage ablations used one, two, or 20 phases; patient-count ablations were performed within both datasets. One cine patient was used for validation and four independent patients were used for testing. Five cardiac structures were evaluated using Dice and the 95th-percentile Hausdorff distance (HD95). Phase-wise and patient-level Dice consistency and structure-specific volumetric variability within and between subjects were assessed.

**Results:**

Full cine training produced higher overall Dice (0.89 vs. 0.87) and lower overall HD95 (4.87 vs. 9.78 mm) than the single-phase reference (both *p* < 0.05). Right ventricular (RV) Dice was higher (0.91 vs. 0.84) and HD95 was lower (5.16 vs. 22.75 mm; both *p* < 0.05). The two-phase model achieved an overall Dice of 0.88 and an overall HD95 of 7.85 mm; neither metric differed significantly from the single-phase reference. HD95 generally increased with smaller cohorts, and both metrics deteriorated markedly in the five-patient configurations. Full cine training showed flatter phase-wise Dice profiles and lower across-phase variability. Between-subject volume differences exceeded phase-related within-subject differences for all structures (all *p* < 0.05).

**Conclusion:**

In this cross-cohort comparison, full cine training showed higher overall Dice, lower overall HD95, and greater phase-wise consistency than single-phase training. The evaluated two-phase configuration may inform annotation-efficient cine CTA dataset design, but broader validation is needed.

## Introduction

Contrast-enhanced cardiac computed tomography angiography (CTA) is widely used for noninvasive assessment of coronary artery disease and cardiac anatomy. Beyond coronary assessment, quantitative analysis of cardiac chambers and myocardium can support measurements of chamber volumes, myocardial mass, and functional indices. Accurate segmentation of cardiac structures is therefore an important prerequisite for reproducible cardiac CTA analysis.

Manual segmentation remains labor-intensive, requires substantial domain expertise, and is subject to inter-observer variability. These limitations become more pronounced for cine CTA, in which each examination is reconstructed at multiple cardiac phases. In the present study, each cine CTA examination contained 20 three-dimensional volumes, making exhaustive manual annotation substantially more demanding than single-phase CTA annotation.

Automated cardiac CTA segmentation is challenging because image appearance varies with contrast timing, radiation dose, slice thickness, field of view, and reconstruction settings. Anatomical factors add further complexity, including cardiac contraction and relaxation, patient-specific chamber morphology, thin myocardial boundaries, and indistinct interfaces between cardiac structures and adjacent tissues.

Our previous work introduced combined multi-atlas and corrective segmentation (CMACS), an automated framework for multi-structure cardiac CTA segmentation (Bui et al., 2020a, 2020b). CMACS enabled large-scale generation of computer-generated labels that were subsequently used to train DeepHeartCT, a 3D convolutional neural network framework for cardiac CTA segmentation (Bui et al., 2022). These studies demonstrated that high-quality computer-generated labels can support scalable deep learning-based segmentation, but they focused primarily on single-phase CTA acquired near mid-diastole.

Automated cardiac CTA segmentation has progressed from multi-atlas registration to deep learning. Kirişli et al. (2010) demonstrated the feasibility of robust chamber segmentation in a large multicenter, multivendor CTA dataset, while later CMACS work improved scalability for heterogeneous CTA data (Bui et al., 2020a, 2020b). U-Net and 3D U-Net architectures established encoder-decoder networks with skip connections for biomedical and volumetric segmentation (Ronneberger et al., 2015; Çiçek et al., 2016), and subsequent cardiac CT studies reported strong performance for chamber, whole-heart, and multi-structure segmentation (Dormer et al., 2018; Baskaran et al., 2020; Bui et al., 2022; Sharkey et al., 2022).

Most cardiac CT segmentation studies have emphasized static or single-phase inputs. By contrast, cine CTA and other four-dimensional cardiac imaging methods provide temporal information relevant to chamber motion, myocardial deformation, and functional analysis (Sun et al., 2024). Bruns et al. (2022) showed that models trained on selected end-systolic and end-diastolic phases can support segmentation across the cardiac cycle in 4D contrast-enhanced cardiac CT. However, prior work has not directly compared one-phase, two-phase, and full-cycle cine training while holding the patient cohort fixed. The effect of reducing the number of unique patients within a given training source also remains incompletely characterized.

Distinguishing total image count, temporal phase coverage, and the number of unique patients is important for medical imaging. Large-scale computer vision and radiology studies suggest that adding samples improves performance most when the additional data broaden the training distribution rather than provide highly correlated examples (Sun et al., 2017; Thian et al., 2022). Dataset redundancy can also bias evaluation and limit generalization in medical imaging benchmarks (Cassidy et al., 2022). Consecutive cine CTA phases are not duplicates, but they are highly correlated samples that share the same patient-specific anatomy, acquisition conditions, and contrast dynamics. Thus, increasing phase coverage and increasing patient cohort size represent distinct approaches to expanding a training dataset.

The present study examines the effects of temporal phase coverage and patient cohort size on deep learning-based cardiac structure segmentation in cine CTA. We trained 3D U-Net models using single-phase and multiphase configurations and evaluated performance in a separate cine CTA test dataset. To assess temporal phase coverage, cine models were trained using one, two, or all 20 phases while holding the 20-patient cine training cohort fixed. To assess patient cohort size, additional models were trained using progressively smaller patient subsets within both the single-phase and cine datasets. The full single-phase and cine configurations were also compared as a cross-cohort benchmark. We additionally quantified structure-specific within-subject phase-related and phase-matched between-subject volumetric variability for different cardiac structures.

The main contributions of this study are a controlled phase-coverage ablation that holds the cine training cohort fixed; within-source patient-count ablations in both datasets; structure-specific comparisons of within- and between-subject volumetric variability; and evaluation of one fixed two-phase configuration as a potential annotation-efficient alternative to full-cycle cine training.

## Materials and methods

All examinations were performed under protocols approved by the Institutional Review Board of the National Institutes of Health. Written informed consent was obtained from all participants before study participation.

### Image acquisition

All CTA studies were acquired on a 320-detector-row scanner at the same institution (Aquilion ONE Genesis, Canon Medical Systems, Japan) with 0.5 mm detector collimation, 275 ms gantry rotation time, 100–120 kVp tube voltage, and 200–850 mA tube current adjusted according to the patient attenuation profile determined from the scout image. Iodinated contrast material (50–70 mL) was administered at 5.0–5.5 mL/s with the dose adjusted for body habitus. Prospective electrocardiography-triggered acquisition began when attenuation in the descending aorta reached 350–400 HU. Images were reconstructed with a 512 × 512 matrix, pixel spacing of 0.26–0.43 mm, 240–520 slices per study, and slice thickness of 0.5–1.0 mm.

### Dataset composition

Two distinct CTA cohorts were included. Both cohorts were broadly comparable with respect to scanner platform, acquisition protocol, and spatial reconstruction parameters, but differed in temporal reconstruction and label-selection procedures.

Cohort 1 comprised 25 patients with cine CTA acquisitions reconstructed in 5% increments throughout the cardiac cycle, yielding 20 phase-specific 3D volumes per patient. Labels for Cohort 1 were initially generated using the CMACS framework. Before model development, five patients were selected from Cohort 1 to provide approximately even coverage of the heart-size distribution and held out from model training. One patient formed the validation-label (VL) dataset (20 volumes), and the remaining four patients formed the independent test-label (TL) dataset (80 volumes).

Reference labels for all five held-out patients underwent expert review and manual correction by an image analyst (A.S.) with four years of experience in cardiac CTA image processing, under the supervision of a cardiologist (M.C.) with more than 20 years of experience in cardiac CTA. Corrections were case-dependent and generally light to moderate, with particular attention to myocardial boundaries. The remaining 20 patients formed the cine-label (CL) training dataset, comprising 400 volumes.

Cohort 2 comprised 1,124 single-phase cardiac CTA scans reconstructed at 75% of the R-R interval near mid-diastole. From this cohort, we used 110 cases previously classified as strong labels (SL) in the DeepHeartCT study. These 110 volumes represented the highest-quality CMACS-generated labels selected by reverse ranking and were used without additional modification (Bui et al., 2022).

### Training configurations and ablations of temporal phase coverage and patient cohort size

Twelve model training configurations were evaluated (Table 1) to examine temporal phase coverage and patient cohort size. Phase coverage was varied within the fixed 20-patient CL cohort, whereas cohort size was reduced separately within the SL and CL datasets. Comparisons between the full SL and CL configurations served as cross-cohort benchmarks.

**Table 1:** Dataset configurations used for model training, validation, and testing.

Dataset	No. of patients	No. of phases	No. of 3D volumes
SL_110n	110	1	110
SL_55n	55	1	55
SL_20n	20	1	20
SL_10n	10	1	10
SL_5n	5	1	5
CL_20n20p	20	20	400
CL_10n20p	10	20	200
CL_20n2p-15&8	20	2	40
CL_20n1p-15	20	1	20
CL_20n1p-8	20	1	20
CL_10n1p-15	10	1	10
CL_5n1p-15	5	1	5
VL	1	20	20
TL	4	20	80

SL: single-phase labels; CL: cine CTA labels; VL: validation labels; TL: test labels. Configuration labels indicate the number of patients (n), phases (p), and 3D training volumes.

The full SL model used 110 single-phase volumes from 110 patients. Additional SL models were trained using subsets of 55, 20, 10, and 5 patients, with one volume per patient. Within the CL cohort, the full CL model used all 20 phases from 20 patients (400 volumes). The two-phase configuration used phases 15 and 8 from each patient (40 volumes), whereas the single-phase configurations used phase-15 or phase-8 from each patient (20 volumes per configuration).

Phase numbers 1–20 corresponded to consecutive 5% increments of the R-R interval. Phase-15 corresponded to 75% of the R-R interval and matched the mid-diastolic reconstruction window used for the SL dataset, whereas phase 8 corresponded to 40% of the R-R interval and represented a contrasting phase near end-systole. In the 25-patient cine cohort, patient-specific end-systolic phases ranged from phases 7 to 10, with a mean phase index of 8.5. Phase 8 was selected before performance evaluation as a representative fixed index within this range. The same fixed phase indices were used for all cine patients, rather than selecting patient-specific end-systolic and end-diastolic phases.

CL cohort-size ablations included a full multiphase model trained on 10 patients using all 20 phases per patient (200 volumes), as well as single-phase models trained using phase-15 in cohorts of 20, 10, and 5 patients (20, 10, and 5 volumes, respectively). All configurations used the same preprocessing, network architecture, and optimization settings, the same VL patient for validation and checkpoint selection, and the same four-patient TL dataset for final evaluation.

The segmentation targets were the left ventricle (LV), right ventricle (RV), left atrium (LA), right atrium (RA), and left ventricular myocardium (LVM). These structures are central to quantitative cardiac assessment and are commonly evaluated in cardiac CT segmentation studies.

### Deep learning framework

All experiments were performed on a workstation equipped with an Intel Core i9-14900F 2.0 GHz CPU, an NVIDIA GeForce RTX 3090 GPU, and 128 GB RAM, using identical parameter settings across configurations. The framework was implemented using Python 3.11.15, PyTorch 2.12.1, CUDA 12.6, and cuDNN 9.1.0. All CTA volumes were resampled to isotropic 1.0 mm^3^ voxels before downstream image processing, model training, and evaluation.

All experiments used the DeepHeartCT framework, a 3D U-Net-based convolutional neural network for cardiac structure segmentation, with the same architecture and parameter settings across training configurations to support controlled comparisons of temporal phase coverage and patient cohort size (Bui et al., 2022). A bounding-box localization step was used to crop the cardiac region before patch extraction, reducing the number of non-cardiac voxels and improving computational efficiency.

The network processed overlapping 64 × 100 × 100 voxel patches with a stride of 32 × 64 × 64 voxels. Each convolutional block consisted of a 3D convolution, rectified linear unit activation, and group normalization. The final convolutional layer was followed by a sigmoid activation to generate probability maps for each target structure. During inference, overlapping patch probabilities were averaged during patch recombination to reconstruct the final 3D segmentation map. A probability threshold of 0.6 was applied to each output channel. When multiple outputs exceeded the threshold at a voxel, labels were assigned according to the following increasing precedence: whole heart, LV, LA, ascending aorta, RA, RV, and LVM. The whole-heart output covered voxels not assigned to a specific evaluated structure; whole heart and ascending aorta were auxiliary outputs and were excluded from the five-structure analysis. Voxels not assigned to a target structure were set to background.

Training used Dice loss, computed separately for each foreground channel and then averaged across channels; the background channel was excluded. The Adam optimizer was used with an initial learning rate of 1 × 10^−4^, weight decay of 1 × 10^−4^, and batch size of 1. Models were initially trained for 20 epochs and extended to a maximum of 150 epochs if validation performance had not converged. Convergence was defined as no improvement in validation performance over 4,000 training batches.

To ensure consistent model selection across configurations, the VL patient was used to monitor convergence and select checkpoints. Checkpoints were saved throughout training, and the checkpoint with the highest validation Dice score was selected for final evaluation. The VL patient was not used for gradient-based model training and was excluded from all reported test performance summaries and comparisons. The same fixed random seed and training settings were used for all configurations. Repeated runs with the same seed were used to assess computational consistency; variability across independent random initializations was not evaluated. No conventional image augmentation was applied so that all configurations were compared under a common training protocol.

### Evaluation metrics

For each trained model, predicted labels were generated for the independent TL dataset and compared with the expert-reviewed reference labels. Segmentation accuracy was quantified using the Dice similarity coefficient and the 95th-percentile Hausdorff distance (HD95) for each cardiac structure across the 80 TL volumes. For each volume and metric, an overall value was calculated as the average of the five structure values. For model summaries, metric values were first averaged across the 20 phases within each TL patient and then summarized across the four patient-level means.

Phase-level performance of the full SL, full CL, and two-phase CL models was summarized as the mean and standard deviation across the four TL patients at each cardiac phase. Patient-level Dice scores were summarized as the mean and standard deviation across the 20 phases within each TL patient for the same three models.

To characterize structure-specific volumetric variability attributable to cardiac motion within subjects and differences between subjects, separate analyses were performed for the volumes of the LV, RV, LA, RA, and LVM in the CL dataset. Structure volumes were calculated from the labeled voxel counts and voxel dimensions and expressed in milliliters (mL).

For each of the 20 CL patients, the volume of each structure was calculated from the CMACS-derived labels at each of the 20 cine phases and used to assess phase-related within-subject variability and phase-matched between-subject variability. The held-out VL and TL patients were excluded from these analyses. The primary within-subject measure summarized average pairwise temporal variation rather than the full end-diastolic-to-end-systolic change.

Additionally, as a range-based sensitivity analysis, the phase-specific volumes for each patient were summarized by the median and observed minimum and maximum. The within-subject range was defined as the maximum minus minimum volume across the 20 phases for each patient, whereas the phase-matched between-subject range was defined at each phase as the maximum minus minimum volume across the 20 CL patients.

These analyses quantified structure-specific volumetric variability and were not intended to characterize broader anatomical differences in cardiac shape or orientation.

### Statistical analysis

Demographic, clinical, and imaging characteristics were compared between the single-phase and multiphase CTA cohorts. Continuous variables were compared using Welch’s independent-samples t-test or the Mann–Whitney U test, as appropriate. Variables unavailable in one cohort or unsuitable for statistical comparison were reported descriptively. Cohort comparison *p* values were adjusted across all estimable comparisons using the Holm correction method (Holm, 1979). Adjusted *p* < 0.05 was considered statistically significant.

Dice scores and HD95 values were analyzed using separate linear mixed-effects models, with training configuration as a fixed effect and subject as a random effect. This approach retained all phase-level observations while accounting for within-subject correlation across the 20 cine phases. Analyses were performed separately for each anatomical structure and for an overall metric, defined as the mean of the five structure-specific values for each volume.

Pairwise comparisons of Dice and HD95 were performed between each training configuration and the full 110-patient SL reference for each cardiac structure and the overall average. Inferential *p* values for both metrics were derived from the corresponding mixed-effects models and adjusted using the Holm method. For each configuration, the reported mean, standard deviation (SD), and t-based 95% confidence interval (CI) half-widths were calculated from four patient-level means.

Additional descriptive analyses were performed for the full SL, full CL, and two-phase CL models to characterize phase-wise and within-patient performance variability. For each cardiac structure, the mean and standard deviation of Dice were calculated across the four TL patients at each of the 20 cardiac phases for the phase-wise analysis and across the 20 cardiac phases within each TL patient for the patient-level analysis.

For the CL volumetric comparison, one within-subject and one between-subject variability measure were calculated for each subject and each cardiac structure to avoid treating repeated phase measurements as independent. Within-subject variability was defined as the mean pairwise absolute difference across the 20 phases, whereas between-subject variability was defined as the mean phase-matched absolute difference between each subject and all other CL subjects at matched phases. The paired primary variability measures and the unpaired range-based measures were compared separately for the LV, RV, LA, RA, and LVM using two-sided Wilcoxon signed-rank and rank-sum tests, respectively; *p* < 0.05 was considered statistically significant.

## Results

Demographic, clinical, and imaging characteristics of the single-phase and multiphase CTA cohorts are summarized in Table 2. Among the characteristics for which statistical comparisons were available, only phase-15 LV cavity volume differed and was lower in the multiphase cohort (*p* = 0.03). Training and inference times for the principal configurations are summarized in Table 3. Training time increased with the number of training volumes. The full 400-volume CL configuration required more than five times the training time of the full 110-volume SL configuration.

**Table 2:** Demographic, clinical, and imaging characteristics of the patient cohorts.

Characteristic	Single-phase CTA cohort (*n* = 110)	Multiphase CTA cohort (*n* = 25)	*p* value (Holm-adjusted)
Sex, female/male	38/72	9/16	
Age (years)	51.5 ± 10.3	52.3 ± 11.6	NS
Height (m)	1.73 ± 0.09	1.70 ± 0.12	NS
Weight (kg)	90.1 ± 16.2	81.2 ± 17.9	NS
Body mass index (kg/m^2^)	30.1 ± 5.1	28.2 ± 6.0	NS
LV volume at phase-15 (mL)	127.2 ± 23.3	111.8 ± 33.8	0.03
LV ejection fraction (%)	Not available	53.6 ± 5.2	
Heart rate (bpm)	56.6 ± 6.2	59.0 ± 7.2	NS
Beta-blocker	Metoprolol	Metoprolol	
Clinical indication	Suspected coronary artery disease	Suspected valvular disease	
Tube voltage (kVp)	100 or 120	100 or 120	
Tube current (mA)	568.7 ± 156.6	499.2 ± 206.9	NS
Exposure time (ms)	275	275	
Tube exposure (mAs)	99.4 ± 27.0	86.9 ± 36.3	NS
Slice thickness (mm)	0.5	0.5 or 1.0	
Pixel spacing (mm)	0.37 ± 0.05	0.36 ± 0.03	NS
Reconstruction diameter (mm)	191.3 ± 26.6	183.0 ± 14.1	NS

Values are presented as mean ± standard deviation or counts, as appropriate. A Holm-adjusted *p* value > 0.05 is indicated as not significant (NS). The multiphase cohort comprises all 25 cine patients in the CL, VL, and TL datasets.

**Table 3:** Available training and inference times across evaluated dataset configurations.

Dataset (volumes)	GPU training time (min)	GPU inference time (sec/volume)	CPU inference time (sec/volume)
SL_110n (110)	40.50	7.69	11.76
CL_20n20p (400)	219.58		
CL_20n2p-15&8 (40)	27.87		

### Qualitative segmentation across cine phases and cardiac views

Figure 1 illustrates cardiac morphology across all 20 phases of a representative TL cine CTA study, together with the corresponding segmentations generated by the full CL model. The model produced visually coherent LV, RV, LA, RA, and LVM labels throughout the cardiac cycle. Figure 2 compares segmentations generated by the full SL, full CL, and two-phase CL models in two TL cases at phases 8 and 15. Results are shown in long-axis two-chamber, three-chamber, and four-chamber views and short-axis views at basal, midventricular, and apical levels, using automated cardiac reorientation and slice extraction (Sun et al., 2026).

**Figure 1:**
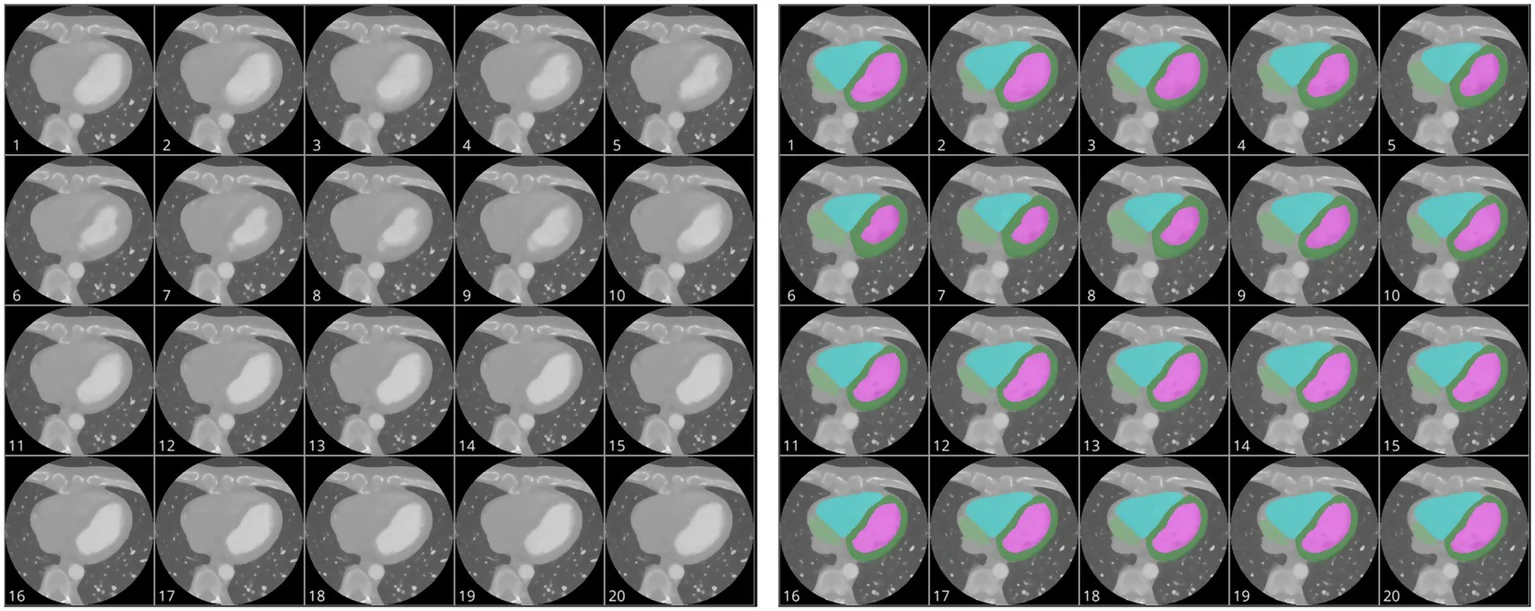
Representative cine CTA images and corresponding cardiac structure segmentations across 20 cardiac phases. A midventricular axial slice is shown for each phase. The left panel displays the source cine CTA images, demonstrating cyclic changes in cardiac morphology, whereas the right panel shows the corresponding segmentations generated by the model trained on the full cine-label dataset. Cardiac phases are arranged in matching positions in both panels. Color labels denote 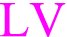, 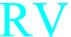, 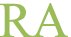, and 
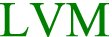
.

**Figure 2:**
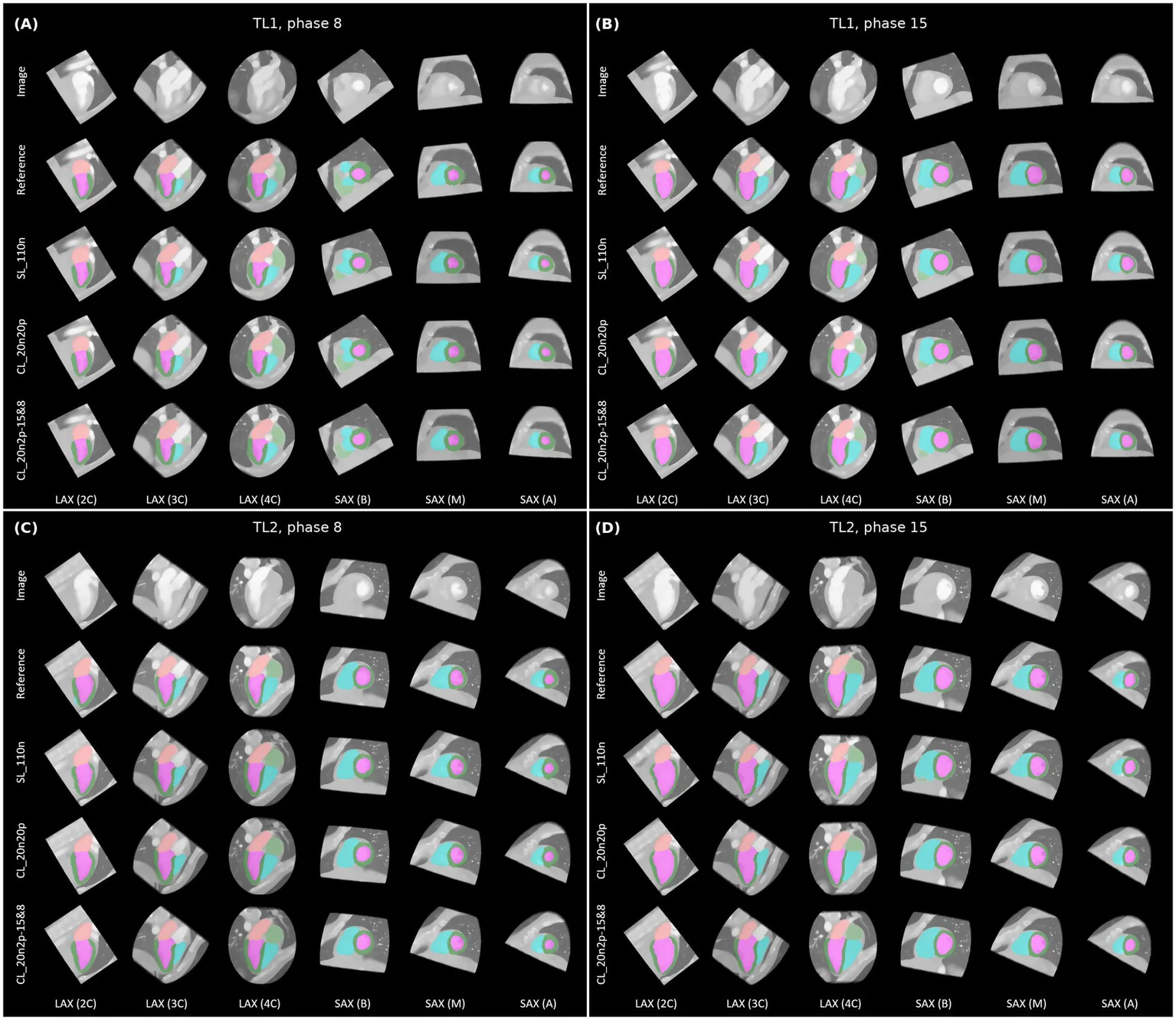
Qualitative comparison of cardiac structure segmentations in two test-label (TL) cases at phases 8 and 15. Panels **(A,B)** show TL1 at phases 8 and 15; panels **(C,D)** show TL2 at phases 8 and 15. In each panel, rows show the source image, expert-corrected reference segmentation, and predictions from the full SL (SL_110n), full CL (CL_20n20p), and two-phase CL (CL_20n2p-15&8) models. Columns show long-axis (LAX) two-chamber (2C), three-chamber (3C), and four-chamber (4C) views and short-axis (SAX) views at the basal (B), midventricular (M), and apical (A) levels. Color labels denote 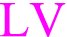, 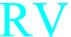, 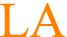, 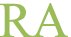, and 
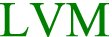
.

### Segmentation performance by training configuration

Figure 3 presents mean Dice scores and HD95 values for the five structures across 12 training configurations, with standard deviations across the four patient-level means. The full 110-patient SL model served as the statistical reference for pairwise comparisons. The corresponding means, standard deviations, and 95% CI for both Dice and HD95 values are provided in Supplementary Tables S1, S2, respectively.

**Figure 3:**
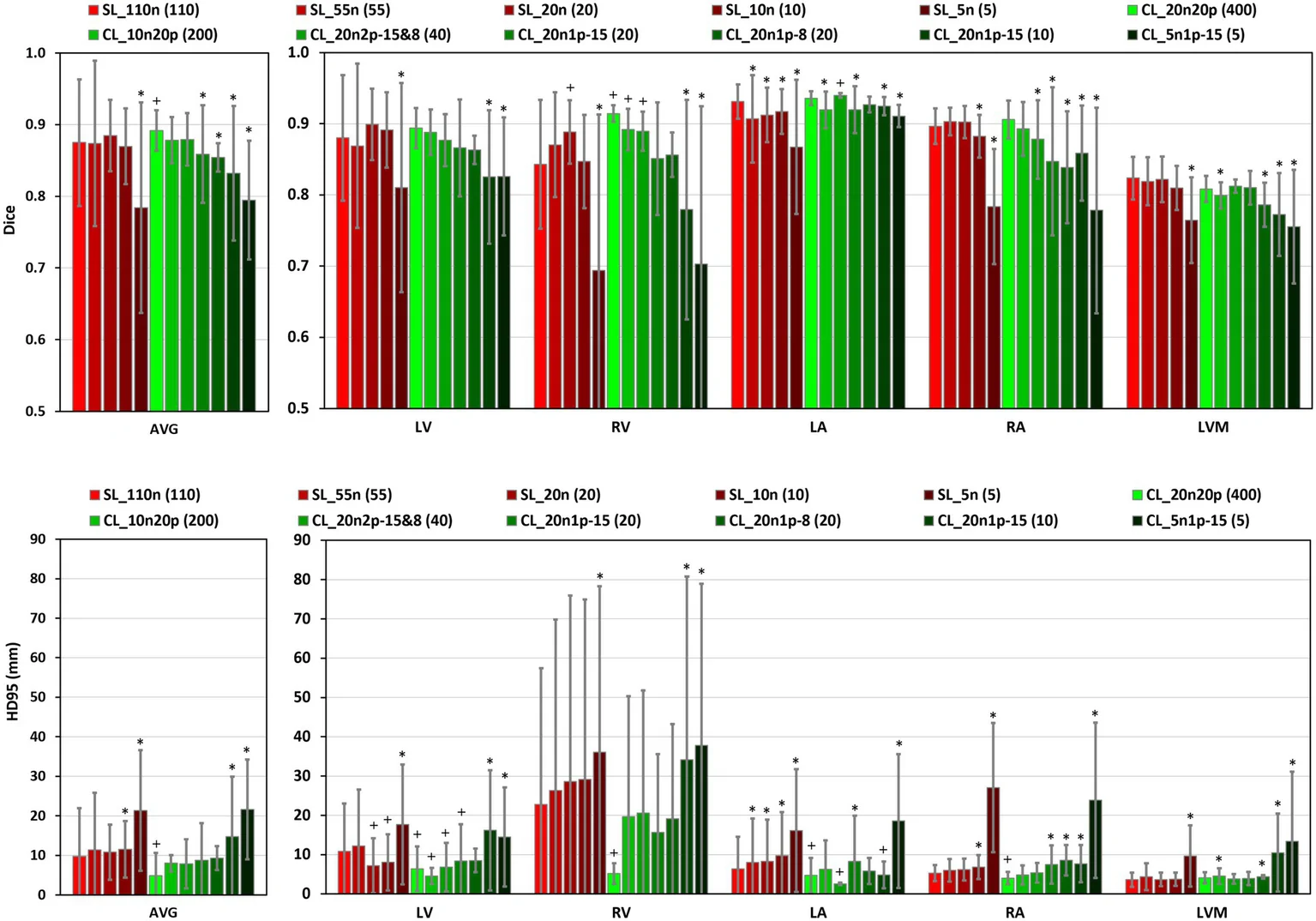
Mean dice scores (top) and mean 95th-percentile Hausdorff distance (HD95) values (bottom) for models trained with 12 dataset configurations. Results are shown for the average (AVG) metric values across the LV, RV, LA, RA, and LVM, as well as for each structure. “+” indicates statistically superior performance (higher Dice or lower HD95; *p* < 0.05) relative to the full SL reference after Holm correction, whereas “*” indicates statistically inferior performance (lower Dice or higher HD95; *p* < 0.05). Error bars represent the standard deviation across the patient-level means.

In the cross-cohort benchmark, the full CL model achieved a higher overall mean Dice than the full SL model (0.89 vs. 0.87, *p* < 0.05) and a lower overall mean HD95 (4.87 vs. 9.78 mm, *p* < 0.05). The corresponding between-patient standard deviations were also smaller with full CL for both Dice (0.01 vs. 0.05) and HD95 (2.92 vs. 11.55 mm).

The clearest structure-specific difference was observed for the RV: the full CL model had higher Dice (0.91 vs. 0.84, *p* < 0.05) and lower HD95 (5.16 vs. 22.75 mm, *p* < 0.05) than the full SL model. Full CL also had lower HD95 for the LV (6.37 vs. 10.88 mm, *p* < 0.05), LA (4.73 vs. 6.39 mm, *p* < 0.05), and RA (4.00 vs. 5.27 mm, *p* < 0.05), but did not differ significantly for the LVM. Dice did not differ significantly for the LV, LA, RA, or LVM.

Within the fixed 20-patient CL cohort, overall mean Dice and HD95 were 0.89 and 4.87 mm with all 20 phases, 0.88 and 7.85 mm with the two-phase model, 0.86 and 8.74 mm with the phase-15 model, and 0.85 and 9.27 mm with the phase-8 model. These descriptive values show higher Dice and lower HD95 with broader phase coverage, with the largest reduction in HD95 occurring between two-phase and full-cycle training. Relative to the full SL reference, the two-phase model did not differ significantly in overall Dice or HD95; it had higher RV and LA Dice and lower LV and LA HD95, but lower RA Dice (all *p* < 0.05). Both phase-8 and phase-15 one-phase models had lower overall Dice than the full SL reference (both *p* < 0.05), whereas neither one-phase model differed significantly in overall HD95 from the reference.

For the patient-count ablation within the SL dataset, overall mean Dice values were 0.87, 0.87, 0.88, 0.87, and 0.78 for cohorts of 110, 55, 20, 10, and 5 patients, respectively. The corresponding mean HD95 values were 9.78, 11.39, 10.79, 11.53, and 21.31 mm. Overall Dice and HD95 remained comparatively stable from 110 to 10 patients. Both metrics deteriorated markedly in the five-patient configuration and were significantly worse than those of the full SL reference (both *p* < 0.05).

Under full 20-phase CL training, reducing cohort size from 20 to 10 patients decreased overall mean Dice from 0.89 to 0.88 and increased overall mean HD95 from 4.87 to 7.99 mm. Under phase-15 training, reducing cohort size from 20 to 10 and 5 patients progressively decreased mean Dice from 0.86 to 0.83 and 0.79, and increased mean HD95 from 8.74 to 14.67 and 21.62 mm. The 10- and 5-patient phase-15 models had lower overall Dice and higher overall HD95 than the full SL reference (all *p* < 0.05).

Overall, marked reductions in training cohort size adversely affected both overlap accuracy and boundary localization, although the magnitude varied by configuration and structure.

Across all configurations, LVM generally had the lowest mean Dice scores, yet its HD95 values were often among the lowest of the five structures. This pattern may reflect the sensitivity of overlap-based metrics such as Dice to small boundary deviations in the thin, non-cavitary myocardium with variable wall thickness. Thus, Dice and HD95 capture complementary aspects of segmentation performance and should be interpreted together.

### Performance across cine phases and patients

Figure 4 shows phase-level mean Dice and standard deviation profiles for each cardiac structure across the four TL patients for the full SL, full CL, and two-phase CL models. The full SL model showed lower mean Dice and greater between-patient variability at several phases, particularly for LV and RV segmentation at phases 8–11. The full CL and two-phase CL models generally exhibited flatter mean profiles and smaller standard deviations across the cardiac cycle, with the full CL model showing the lowest average variability. Averaged across all 20 phases and five structures, the mean phase-specific standard deviation across patients was 0.06 for the full SL model, 0.02 for the full CL model, and 0.03 for the two-phase CL model. Across phases 8–11, the corresponding values were 0.11, 0.03, and 0.04, respectively.

**Figure 4:**
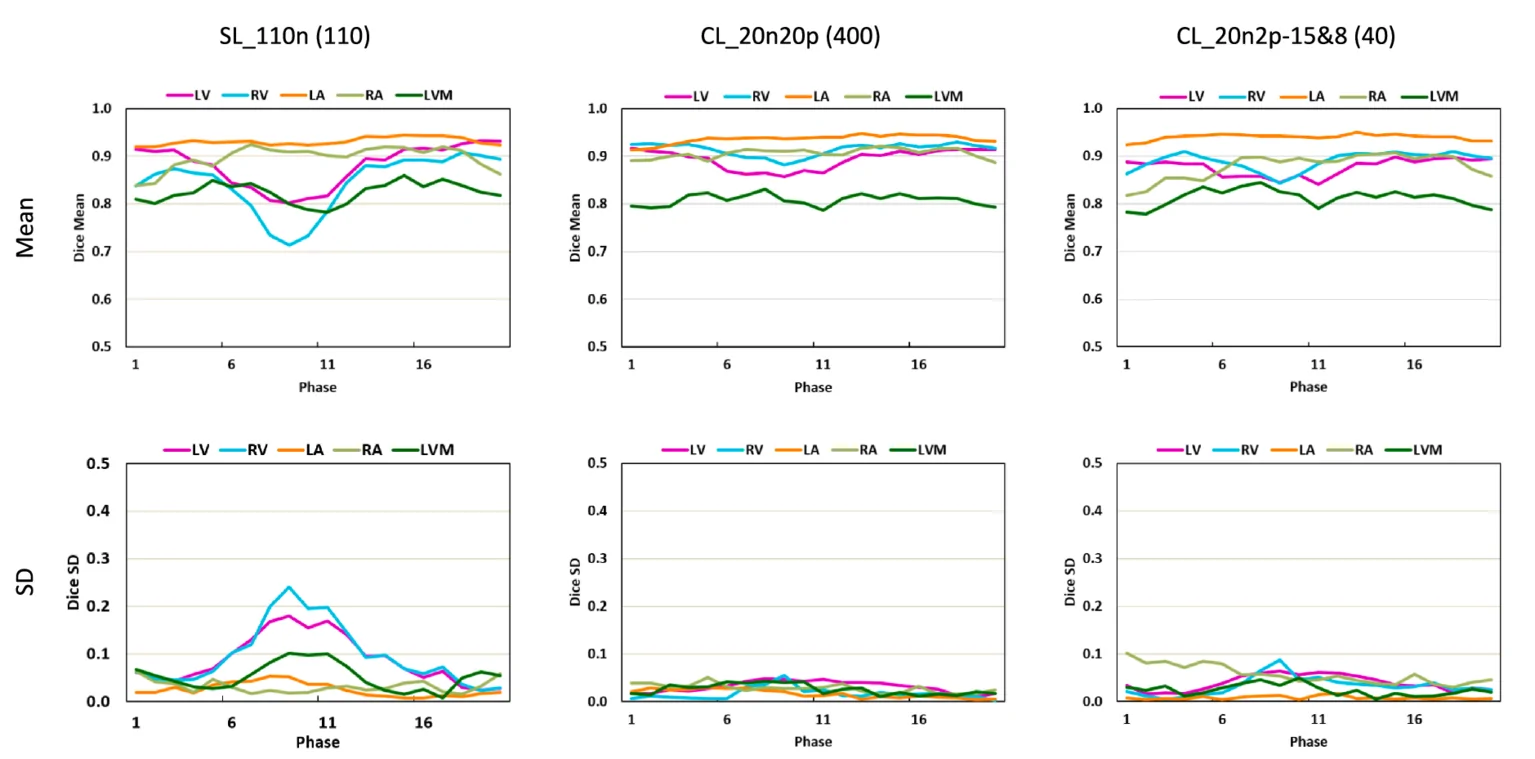
Phase-level dice performance of the full SL (SL_110n), full CL (CL_20n20p), and two-phase CL (CL_20n2p-15&8) models for five structures across 20 cine CTA phases. The top row shows the mean across patients at each phase, and the bottom row shows the corresponding standard deviation across patients. The full SL model showed greater variability across patients at several phases, particularly at phases 8–11, approximately spanning end-systole to early diastole. The full CL and two-phase CL models generally showed flatter mean profiles and smaller standard deviations than the full SL model across the cardiac cycle, with the full CL model showing the lowest average variability.

Figure 5 shows each TL patient’s mean Dice and standard deviation across 20 phases for the full SL, full CL, and two-phase CL models. The full CL and two-phase CL models generally showed smaller within-patient standard deviations across phases than the full SL model, with the full CL model showing marginally lower average variability than the two-phase CL model before rounding. Averaged across the four TL patients and five structures, the mean within-patient standard deviation was 0.04 for the full SL model and 0.02 for both the full CL and two-phase CL models. However, no model was uniformly superior across all structures, phases, and patients. The phase-wise and patient-level values underlying Figures 4, 5 are provided in Supplementary Tables S3, S4, respectively.

**Figure 5:**
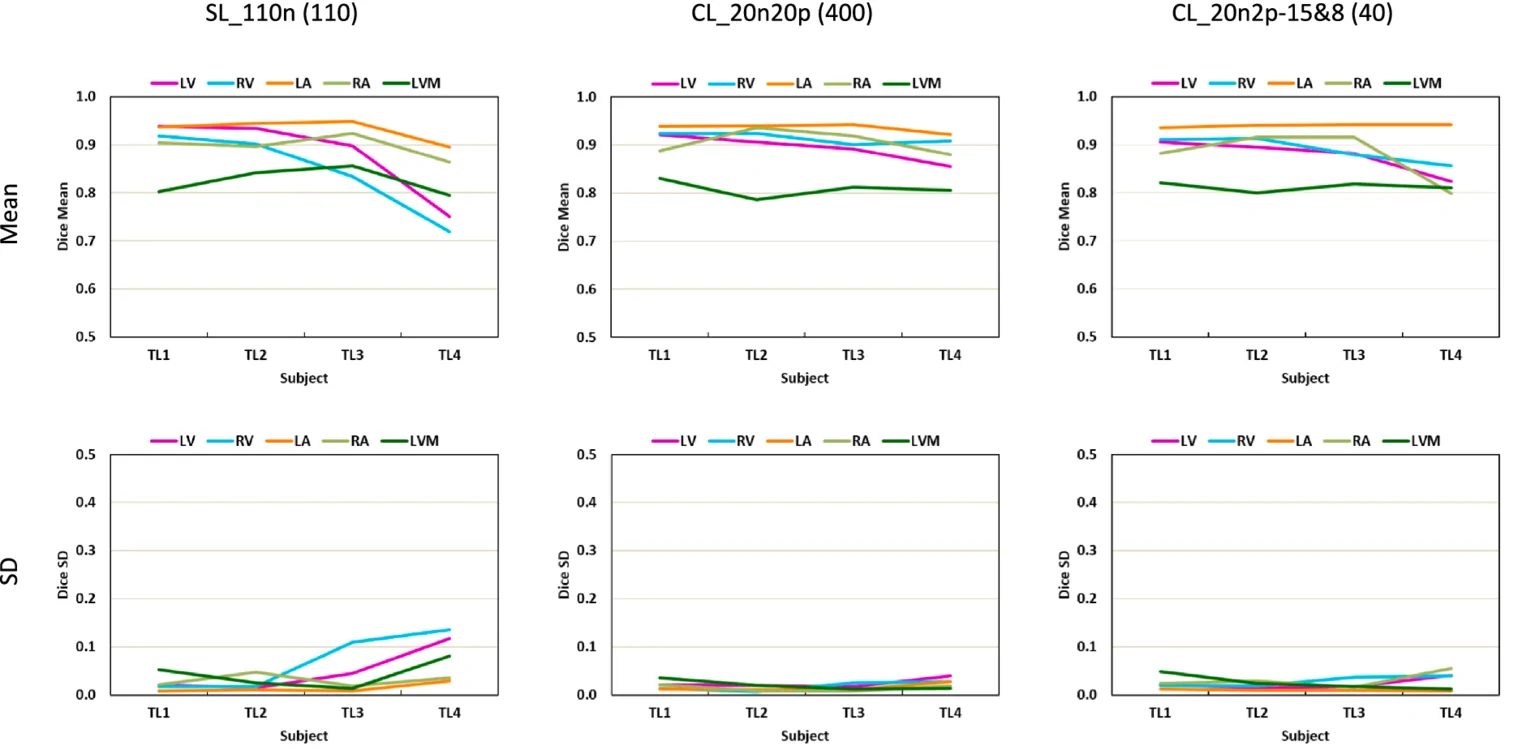
Patient-level dice performance of the full SL (SL_110n), full CL (CL_20n20p), and two-phase CL (CL_20n2p-15&8) models for five structures across the four TL patients. The top row shows each patient’s mean across 20 phases, and the bottom row shows the corresponding within-patient standard deviation. The full CL and two-phase CL models generally showed smaller within-patient variability across phases than the full SL model.

### Within-subject versus between-subject volumetric variability

Across the CL dataset, the volumes of the LV, RV, LA, RA, and LVM showed greater between-subject variability than within-subject phase-related variability. Figure 6 demonstrates larger differences across subject columns than across phases within individual subjects. Figure 7 similarly shows broad differences in mean structure volumes between subjects and smaller variation across phases within subjects. Phase-matched between-subject mean absolute volume differences were significantly greater than within-subject phase-related differences for all five structures (all *p* < 0.05). The range-based analysis also showed greater phase-matched ranges across subjects than within-subject max–min volume ranges for all five structures (all *p* < 0.05; Supplementary Figure S1). These analyses characterize structure-specific volumetric variability rather than broader anatomical diversity.

**Figure 6:**
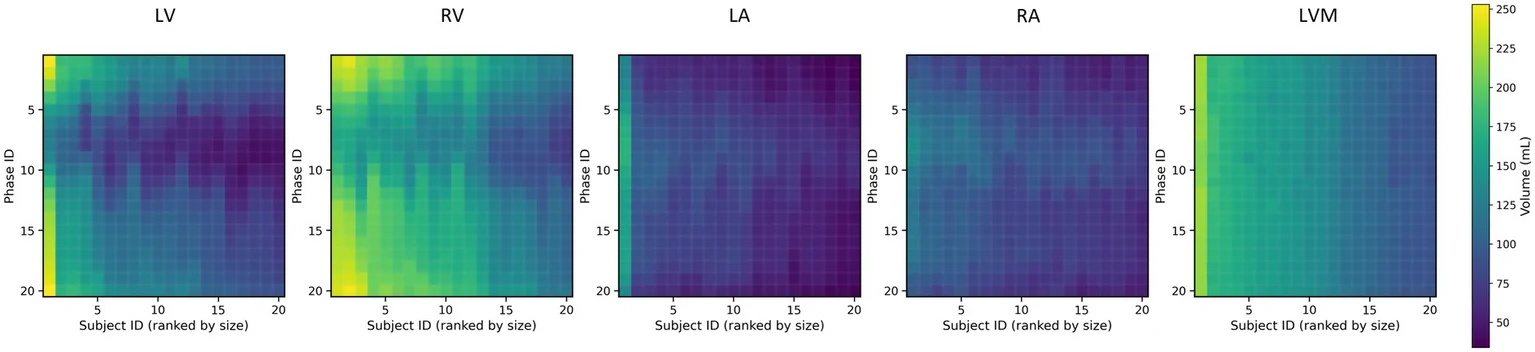
Heatmaps of LV, RV, LA, RA, and LVM volumes across 20 cardiac phases in the 20 CL patients. Within each panel, subjects are ranked from largest to smallest mean volume across phases. Between-subject differences (across columns) were greater than phase-related changes (across rows) within individual subject columns.

**Figure 7:**
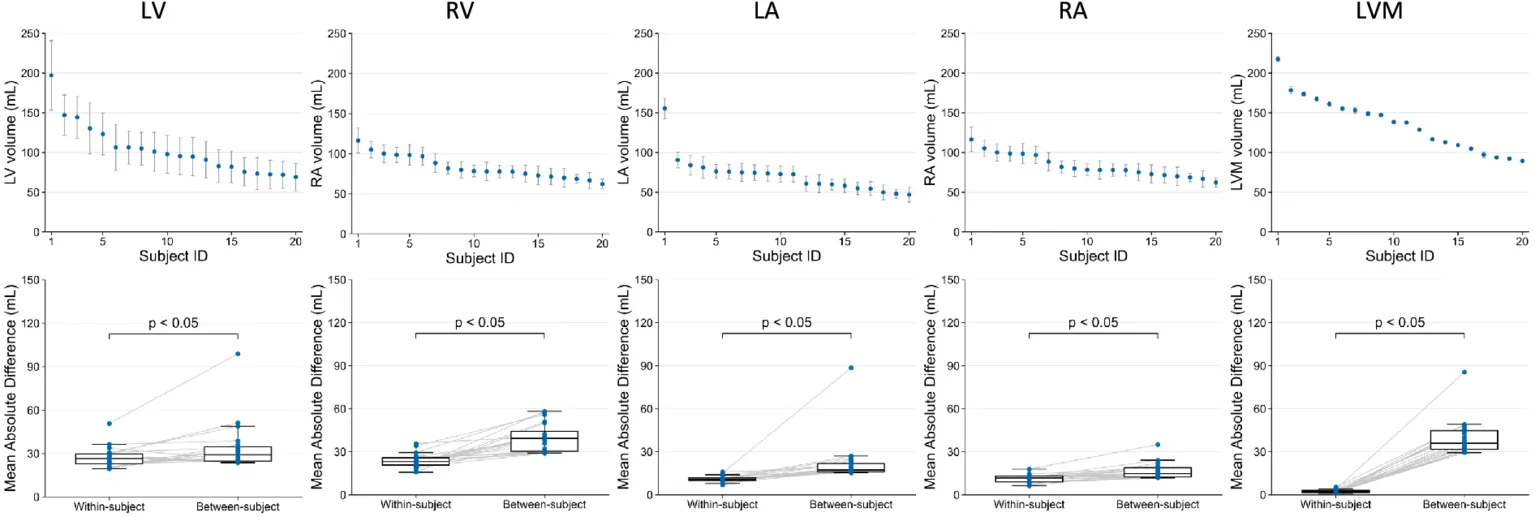
Within-subject versus between-subject variability in LV, RV, LA, RA, and LVM volumes in the CL dataset. Top row: subject-level mean volumes with standard deviations across cardiac phases, showing substantial between-subject differences and comparatively smaller phase-related variation within subjects. Bottom row: box plots of subject-level mean absolute volume differences comparing within-subject variability across phases with phase-matched between-subject variability. Between-subject variability was significantly greater than within-subject variability for all structures (all *p* < 0.05).

## Discussion

This study evaluated how temporal phase coverage and patient cohort size affect deep learning-based cardiac structure segmentation in cine CTA. Five main findings emerged.

1. In the cross-cohort benchmark, full 20-phase CL training yielded higher overall Dice and lower overall HD95 than the 110-patient SL reference, with the clearest structure-specific benefit in the RV.
2. Within the fixed 20-patient CL cohort, the two-phase model had slightly lower mean Dice and higher mean HD95 than the full 20-phase model, whereas both one-phase models had lower Dice and higher HD95 than the two-phase model.
3. Phase-wise and patient-level analyses showed flatter Dice profiles and lower average variability for the full CL and two-phase CL models than for the full SL model, with the full CL model generally showing the lowest variability.
4. Within-source patient-count ablations showed that HD95 generally increased as training cohort size decreased, while both Dice and HD95 deteriorated markedly in the five-patient configurations.
5. Both phase-matched between-subject volumetric variability and volume ranges exceeded the corresponding within-subject phase-related measures for all five structures, indicating greater structure-specific volume differences between patients than across phases within patients.

The greater phase-wise consistency observed with full CL training may reflect exposure to physiologically structured variation across the cardiac cycle. Unlike random intensity or geometric augmentation, cine CTA provides physiologically plausible changes in cardiac anatomy during contraction and relaxation. Exposure to this temporal variation may help the model learn more phase-consistent representations of chamber size, shape, and spatial relationships. In the phase-wise evaluation, the full CL model showed flatter mean Dice profiles and smaller standard deviations across the four TL patients than the full SL model, particularly at phases 8–11 (Figure 4). The patient-level analysis likewise showed generally smaller standard deviations across 20 phases for each patient with the full CL model (Figure 5). Although overall Dice was significantly higher with the full CL model, the cross-cohort design precludes attributing this difference solely to temporal phase coverage. The phase-wise and patient-level analyses more directly support greater temporal consistency across the cardiac cycle, while the RV showed the clearest structure-specific benefit.

The fixed-cohort phase-coverage ablation further supports a contribution from temporal sampling. Increasing coverage from one phase to the evaluated pair of phases 8 and 15 increased the mean Dice score, whereas increasing coverage further to all 20 phases produced only a modest additional increase in Dice but a more pronounced reduction in overall mean HD95. The two-phase CL configuration used 40 training volumes, one-tenth of the 400 phase-level volumes used for full cine training, and achieved a mean Dice score of 0.88 and an overall mean HD95 of 7.85 mm, compared with 0.89 and 4.87 mm for the full CL model. These descriptive differences suggest an additional benefit of full-cycle phase coverage for boundary accuracy. However, the numerical proximity in mean Dice should not be interpreted as statistical equivalence, nor should the findings be generalized to other two-phase annotation strategies.

The patient-count ablations provide more direct evidence regarding patient cohort size. Within the SL dataset, overall Dice remained comparatively stable as the cohort decreased from 110 to 55, 20, and 10 patients, but mean HD95 generally increased. Both metrics deteriorated markedly when only five patients were used. Within the CL dataset, reducing full 20-phase training from 20 to 10 patients modestly reduced Dice and increased HD95, whereas reducing the one-phase CL training cohort at phase-15 from 20 to 10 and five patients produced progressively poorer Dice and HD95 values. These findings suggest that boundary accuracy may deteriorate before a large change becomes evident in Dice and that very small patient cohorts are particularly vulnerable to performance loss. They do not, however, establish a universal minimum number of training patients, because the observed effects may depend on patient composition, label quality, acquisition domain, and training protocol.

The within-source ablations should be distinguished from the direct comparison of the full SL and full CL cohorts. Data for both cohorts were acquired at the same institution using the same scanner platform and broadly comparable acquisition and spatial reconstruction protocols. Most available characteristics for statistical comparison did not differ significantly, but the cohorts differed in phase-15 LV cavity volume, temporal reconstruction, clinical indication, and label-selection procedures. Consequently, the observed full-CL advantage cannot be attributed solely to temporal phase coverage or patient cohort size. The within-source patient-count and fixed-cohort phase-coverage ablations provide more controlled complementary evidence but do not eliminate residual confounding in the direct cross-cohort benchmark.

These findings are consistent with broader observations that dataset size alone does not guarantee improved generalization when added samples are distributionally redundant. Large-scale deep learning studies suggest that performance gains depend not only on sample count but also on informative variability (Sun et al., 2017). Similar diminishing returns have been reported in radiology tasks as training volume increases (Thian et al., 2022), and benchmark analyses have shown that duplicate or near-duplicate images can bias model development and evaluation (Cassidy et al., 2022). Cine CTA phases are not duplicates, but adjacent phases remain correlated observations from the same patient, acquisition, and contrast administration. The present findings therefore support treating total image count, temporal phase coverage, and the number of unique patients as related but distinct dataset-design factors.

The results also highlight structure-specific differences. RV segmentation showed the clearest benefit from full cine training, which may reflect its complex phase-dependent changes in shape and contrast across the cardiac cycle. By contrast, neither Dice nor HD95 differed significantly between the full SL and full CL models for LVM. Across configurations, LVM generally had the lowest Dice scores but also the lowest HD95 values among the five structures. This contrast underscores the complementary behavior of overlap- and boundary-based metrics when evaluating the thin, tapered myocardial wall, a three-dimensional structure bounded by the endocardial and epicardial surfaces. Small deviations at either surface can substantially reduce spatial overlap while producing comparatively small surface-distance errors. Myocardial segmentation may also be affected by motion-related blurring at the myocardium-blood pool interface, papillary muscle labeling, and training-label quality. Therefore, dedicated boundary-aware losses, higher-resolution models, or explicit temporal architectures could be evaluated in future work.

This study has several limitations.

- The cine training cohort included only 20 patients; one additional patient was used for validation, and the test dataset comprised only four patients, limiting the precision of performance estimates and subgroup analyses.
- All data were obtained at a single institution using a single scanner platform and broadly similar acquisition protocols; external validation across scanners, vendors, reconstruction methods, and patient populations is therefore needed.
- Although within-source patient-count ablations were performed, comparisons between SL- and CL-trained models remained cross-cohort benchmarks subject to residual cohort differences. Variability across alternative patient-subset compositions was also not evaluated.
- Only one fixed two-phase combination was evaluated using the same phase indices for all patients rather than patient-specific end-systolic and end-diastolic phases; generalizability to other phase pairs and to patients with irregular cardiac rhythms remains uncertain.
- The CMACS-derived CL labels may contain residual errors affecting model training and volume measurements, although the TL labels were manually corrected. The volumetric analyses assessed structure size but not other morphologic features and did not validate the observed extrema as physiologic end-diastole or end-systole.
- The study used a single 3D U-Net architecture with fixed hyperparameters, a common random seed, and one fixed VL patient for validation. Conventional image augmentation was not applied, alternative temporal architectures were not evaluated, and variability across independent random initializations was not assessed.
- Model-derived functional measures, including LV end-diastolic and end-systolic volumes, stroke volume, and ejection fraction, were not evaluated. Future work could assess whether the resulting segmentations support accurate and reproducible estimation of these measures.

## Conclusion

In the cross-cohort comparison, full-cycle 20-phase cine training yielded higher overall Dice, lower overall HD95, and greater phase-wise consistency than the larger single-phase reference, with the clearest structure-specific benefit for the RV. These findings remain subject to residual cohort differences and limited precision from only four independent test patients. The evaluated two-phase configuration required substantially fewer phase-level annotations but had slightly lower mean Dice and higher overall mean HD95 than the full 20-phase training. Boundary performance generally worsened as training cohort size decreased, and both overlap and boundary performance deteriorated markedly in the smallest cohorts. Between-subject volumetric variability exceeded phase-related within-subject variability across all structures. These findings support jointly considering temporal phase coverage and patient cohort size in cine CTA dataset design, with broader validation needed before generalizing the selected phase pair or defining a minimum training-cohort size.

## Data Availability

The datasets analyzed in this study are not publicly available because they contain patient imaging data subject to confidentiality and institutional review board restrictions. Requests to access the datasets should be directed to the corresponding author and will be considered subject to applicable approvals and data-use agreements. Requests to access these datasets should be directed to lyhsu@nih.gov.
