# Supplementary material for: Temporal phase coverage and patient cohort size in deep learning-based cardiac cine computed tomography angiography segmentation
Source: Front Artif Intell. 2026 Oct 5;9:1914255. doi: 10.3389/frai.2026.1914255 (PMC13677920; doi:10.3389/frai.2026.1914255)
Supplement: Supplementary file 1 [file Data_Sheet_1.docx]

| LV | RV | LA | RA | LVM |
| --- | --- | --- | --- | --- |
|  |  |  |  |  |
|  |  |  |  |  |

**Supplementary Figure S1.** Range-based within-subject and between-subject volumetric variability in the CL dataset. Top row: median LV, RV, LA, RA, and LVM volumes for each CL patient, with bars spanning the observed minimum and maximum across 20 cardiac phases; patients are ordered by median volume. Bottom row: within-subject maximum-to-minimum ranges across phases for each patient and phase-matched between-subject ranges across the 20 CL patients. Points represent patients or phases, center bars indicate medians, and whiskers span the observed minimum and maximum. Between-subject ranges were greater than within-subject ranges for all structures (all p < 0.05).
