# Supplementary material for: Temporal phase coverage and patient cohort size in deep learning-based cardiac cine computed tomography angiography segmentation
Source: Front Artif Intell. 2026 Oct 5;9:1914255. doi: 10.3389/frai.2026.1914255 (PMC13677920; doi:10.3389/frai.2026.1914255)
Supplement: Supplementary file 2 [file Table_1.docx]

**Supplementary Table S1.** Mean Dice scores, standard deviations (SD), and 95% confidence intervals (CI) half-widths for the 12 training configurations shown in Figure 3.

| **Mean Dice** | | | | | | |
| --- | --- | --- | --- | --- | --- | --- |
| **Configuration** | **AVG** | **LV** | **RV** | **LA** | **RA** | **LVM** |
| SL_110n (110) | 0.87 | 0.88 | 0.84 | 0.93 | 0.90 | 0.82 |
| SL_55n (55) | 0.87 | 0.87 | 0.87 | 0.91 | 0.90 | 0.82 |
| SL_20n (20) | 0.88 | 0.90 | 0.89 | 0.91 | 0.90 | 0.82 |
| SL_10n (10) | 0.87 | 0.89 | 0.85 | 0.92 | 0.88 | 0.81 |
| SL_5n (5) | 0.78 | 0.81 | 0.69 | 0.87 | 0.78 | 0.76 |
| CL_20n20p (400) | 0.89 | 0.89 | 0.91 | 0.94 | 0.91 | 0.81 |
| CL_10n20p (200) | 0.88 | 0.89 | 0.89 | 0.92 | 0.89 | 0.80 |
| CL_20n2p-15&8 (40) | 0.88 | 0.88 | 0.89 | 0.94 | 0.88 | 0.81 |
| CL_20n1p-15 (20) | 0.86 | 0.87 | 0.85 | 0.92 | 0.85 | 0.81 |
| CL_20n1p-8 (20) | 0.85 | 0.86 | 0.86 | 0.93 | 0.84 | 0.79 |
| CL_10n1p-15 (10) | 0.83 | 0.83 | 0.78 | 0.92 | 0.86 | 0.77 |
| CL_5n1p-15 (5) | 0.79 | 0.83 | 0.70 | 0.91 | 0.78 | 0.76 |
|  | | | | | | |
| **SD** \| \| \| \| \| \|; **Configuration** \| **AVG** \| **LV** \| **RV** \| **LA** \| **RA** \| **LVM**; SL_110n (110) \| 0.05 \| 0.09 \| 0.09 \| 0.02 \| 0.02 \| 0.03; SL_55n (55) \| 0.06 \| 0.12 \| 0.07 \| 0.06 \| 0.02 \| 0.03; SL_20n (20) \| 0.04 \| 0.05 \| 0.04 \| 0.04 \| 0.02 \| 0.03; SL_10n (10) \| 0.04 \| 0.05 \| 0.07 \| 0.03 \| 0.03 \| 0.03; SL_5n (5) \| 0.12 \| 0.15 \| 0.22 \| 0.09 \| 0.08 \| 0.06; CL_20n20p (400) \| 0.02 \| 0.03 \| 0.01 \| 0.01 \| 0.03 \| 0.02; CL_10n20p (200) \| 0.03 \| 0.03 \| 0.03 \| 0.03 \| 0.04 \| 0.02; CL_20n2p-15&8 (40) \| 0.03 \| 0.04 \| 0.03 \| 0.00 \| 0.06 \| 0.01; CL_20n1p-15 (20) \| 0.06 \| 0.07 \| 0.08 \| 0.03 \| 0.10 \| 0.02; CL_20n1p-8 (20) \| 0.03 \| 0.02 \| 0.03 \| 0.01 \| 0.08 \| 0.03; CL_10n1p-15 (10) \| 0.08 \| 0.09 \| 0.15 \| 0.01 \| 0.07 \| 0.06; CL_5n1p-15 (5) \| 0.11 \| 0.08 \| 0.22 \| 0.02 \| 0.14 \| 0.08 | | | | | | |
| **Configuration** | **AVG** | **LV** | **RV** | **LA** | **RA** | **LVM** |
| SL_110n (110) | 0.05 | 0.09 | 0.09 | 0.02 | 0.02 | 0.03 |
| SL_55n (55) | 0.06 | 0.12 | 0.07 | 0.06 | 0.02 | 0.03 |
| SL_20n (20) | 0.03 | 0.05 | 0.04 | 0.04 | 0.02 | 0.03 |
| SL_10n (10) | 0.03 | 0.05 | 0.07 | 0.03 | 0.03 | 0.03 |
| SL_5n (5) | 0.11 | 0.15 | 0.22 | 0.09 | 0.08 | 0.06 |
| CL_20n20p (400) | 0.01 | 0.03 | 0.01 | 0.01 | 0.03 | 0.02 |
| CL_10n20p (200) | 0.02 | 0.03 | 0.03 | 0.03 | 0.04 | 0.02 |
| CL_20n2p-15&8 (40) | 0.02 | 0.04 | 0.03 | 0.00 | 0.06 | 0.01 |
| CL_20n1p-15 (20) | 0.06 | 0.07 | 0.08 | 0.03 | 0.10 | 0.02 |
| CL_20n1p-8 (20) | 0.02 | 0.02 | 0.03 | 0.01 | 0.08 | 0.03 |
| CL_10n1p-15 (10) | 0.07 | 0.09 | 0.15 | 0.01 | 0.07 | 0.06 |
| CL_5n1p-15 (5) | 0.10 | 0.08 | 0.22 | 0.02 | 0.14 | 0.08 |
|  | | | | | | |
| **95% CI** | | | | | | |
| **Configuration** | **AVG** | **LV** | **RV** | **LA** | **RA** | **LVM** |
| SL_110n (110) | 0.08 | 0.14 | 0.14 | 0.04 | 0.04 | 0.05 |
| SL_55n (55) | 0.09 | 0.18 | 0.12 | 0.10 | 0.03 | 0.05 |
| SL_20n (20) | 0.05 | 0.08 | 0.07 | 0.06 | 0.04 | 0.05 |
| SL_10n (10) | 0.06 | 0.08 | 0.10 | 0.05 | 0.05 | 0.05 |
| SL_5n (5) | 0.17 | 0.23 | 0.35 | 0.15 | 0.13 | 0.10 |
| CL_20n20p (400) | 0.02 | 0.04 | 0.02 | 0.02 | 0.04 | 0.03 |
| CL_10n20p (200) | 0.04 | 0.05 | 0.05 | 0.04 | 0.06 | 0.03 |
| CL_20n2p-15&8 (40) | 0.04 | 0.06 | 0.04 | 0.00 | 0.09 | 0.01 |
| CL_20n1p-15 (20) | 0.09 | 0.11 | 0.13 | 0.05 | 0.17 | 0.04 |
| CL_20n1p-8 (20) | 0.02 | 0.03 | 0.05 | 0.02 | 0.13 | 0.05 |
| CL_10n1p-15 (10) | 0.12 | 0.15 | 0.25 | 0.02 | 0.11 | 0.09 |
| CL_5n1p-15 (5) | 0.16 | 0.13 | 0.35 | 0.02 | 0.23 | 0.13 |

**Supplementary Table S2.** Mean HD95 values, standard deviations (SD), and 95% confidence intervals (CI) half-widths for the 12 training configurations shown in Figure 3.

| **Mean HD95** | | | | | | |
| --- | --- | --- | --- | --- | --- | --- |
| **Configuration** | **AVG** | **LV** | **RV** | **LA** | **RA** | **LVM** |
| SL_110n (110) | 9.78 | 10.88 | 22.75 | 6.39 | 5.27 | 3.60 |
| SL_55n (55) | 11.39 | 12.20 | 26.31 | 8.03 | 6.02 | 4.40 |
| SL_20n (20) | 10.79 | 7.21 | 28.60 | 8.25 | 6.20 | 3.67 |
| SL_10n (10) | 11.53 | 8.06 | 29.18 | 9.78 | 6.83 | 3.78 |
| SL_5n (5) | 21.31 | 17.69 | 36.02 | 16.13 | 27.05 | 9.65 |
| CL_20n20p (400) | 4.87 | 6.37 | 5.16 | 4.73 | 4.00 | 4.09 |
| CL_10n20p (200) | 7.99 | 4.57 | 19.70 | 6.31 | 4.83 | 4.51 |
| CL_20n2p-15&8 (40) | 7.85 | 6.83 | 20.63 | 2.57 | 5.38 | 3.82 |
| CL_20n1p-15 (20) | 8.74 | 8.36 | 15.66 | 8.28 | 7.45 | 3.95 |
| CL_20n1p-8 (20) | 9.27 | 8.51 | 19.09 | 5.83 | 8.59 | 4.34 |
| CL_10n1p-15 (10) | 14.67 | 16.18 | 34.19 | 4.81 | 7.68 | 10.51 |
| CL_5n1p-15 (5) | 21.62 | 14.50 | 37.82 | 18.57 | 23.84 | 13.37 |
|  | | | | | | |
| **SD** | | | | | | |
| **Configuration** | **AVG** | **LV** | **RV** | **LA** | **RA** | **LVM** |
| SL_110n (110) | 11.55 | 12.12 | 34.67 | 8.17 | 2.05 | 1.81 |
| SL_55n (55) | 14.87 | 14.41 | 43.50 | 11.14 | 2.86 | 3.40 |
| SL_20n (20) | 13.74 | 7.00 | 47.30 | 10.71 | 2.78 | 1.72 |
| SL_10n (10) | 13.09 | 7.13 | 45.76 | 11.00 | 3.04 | 1.69 |
| SL_5n (5) | 18.81 | 15.26 | 42.31 | 15.59 | 16.42 | 7.79 |
| CL_20n20p (400) | 2.92 | 5.70 | 2.62 | 4.40 | 1.59 | 1.43 |
| CL_10n20p (200) | 8.78 | 2.06 | 30.60 | 7.32 | 2.45 | 1.98 |
| CL_20n2p-15&8 (40) | 8.11 | 6.20 | 31.15 | 0.32 | 2.51 | 1.20 |
| CL_20n1p-15 (20) | 9.31 | 9.35 | 19.96 | 11.60 | 4.87 | 1.67 |
| CL_20n1p-8 (20) | 5.37 | 3.03 | 24.14 | 3.31 | 3.86 | 0.48 |
| CL_10n1p-15 (10) | 15.67 | 15.27 | 46.56 | 3.43 | 4.73 | 9.92 |
| CL_5n1p-15 (5) | 20.85 | 12.61 | 41.12 | 17.00 | 19.73 | 17.72 |
|  | | | | | | |
| **95% CI** | | | | | | |
| **Configuration** | **AVG** | **LV** | **RV** | **LA** | **RA** | **LVM** |
| SL_110n (110) | 18.38 | 19.29 | 55.17 | 12.99 | 3.27 | 2.88 |
| SL_55n (55) | 23.66 | 22.93 | 69.22 | 17.72 | 4.56 | 5.41 |
| SL_20n (20) | 21.86 | 11.13 | 75.26 | 17.04 | 4.43 | 2.73 |
| SL_10n (10) | 20.82 | 11.35 | 72.81 | 17.51 | 4.84 | 2.70 |
| SL_5n (5) | 29.93 | 24.29 | 67.33 | 24.81 | 26.12 | 12.40 |
| CL_20n20p (400) | 4.64 | 9.07 | 4.17 | 7.00 | 2.53 | 2.27 |
| CL_10n20p (200) | 13.97 | 3.28 | 48.70 | 11.65 | 3.90 | 3.16 |
| CL_20n2p-15&8 (40) | 12.91 | 9.87 | 49.56 | 0.52 | 4.00 | 1.92 |
| CL_20n1p-15 (20) | 14.81 | 14.88 | 31.76 | 18.46 | 7.75 | 2.67 |
| CL_20n1p-8 (20) | 8.55 | 4.82 | 38.42 | 5.27 | 6.15 | 0.76 |
| CL_10n1p-15 (10) | 24.93 | 24.29 | 74.08 | 5.45 | 7.52 | 15.78 |
| CL_5n1p-15 (5) | 33.18 | 20.07 | 65.43 | 27.06 | 31.39 | 28.19 |

**Supplementary Table S3.** Phase-level Dice values for the full SL, full CL, and two-phase CL models across 20 cine CTA phases, corresponding to Figure 4. For each phase, the mean and standard deviation were calculated across the four independent TL patients.

| **Mean** | **SL_110n** | | | | | **CL_20n20p** | | | | | **CL_20n2p-15&8** | | | | |
| --- | --- | --- | --- | --- | --- | --- | --- | --- | --- | --- | --- | --- | --- | --- | --- |
| **Phase** | **LV** | **RV** | **LA** | **RA** | **LVM** | **LV** | **RV** | **LA** | **RA** | **LVM** | **LV** | **RV** | **LA** | **RA** | **LVM** |
| 1 | 0.91 | 0.84 | 0.92 | 0.84 | 0.81 | 0.92 | 0.93 | 0.91 | 0.89 | 0.80 | 0.89 | 0.86 | 0.92 | 0.82 | 0.78 |
| 2 | 0.91 | 0.86 | 0.92 | 0.84 | 0.80 | 0.91 | 0.93 | 0.92 | 0.89 | 0.79 | 0.88 | 0.88 | 0.93 | 0.82 | 0.78 |
| 3 | 0.91 | 0.87 | 0.93 | 0.88 | 0.82 | 0.91 | 0.92 | 0.92 | 0.90 | 0.79 | 0.89 | 0.90 | 0.94 | 0.85 | 0.80 |
| 4 | 0.89 | 0.86 | 0.93 | 0.89 | 0.82 | 0.90 | 0.92 | 0.93 | 0.90 | 0.82 | 0.88 | 0.91 | 0.94 | 0.85 | 0.82 |
| 5 | 0.88 | 0.86 | 0.93 | 0.88 | 0.85 | 0.90 | 0.92 | 0.94 | 0.89 | 0.82 | 0.88 | 0.90 | 0.94 | 0.85 | 0.84 |
| 6 | 0.84 | 0.83 | 0.93 | 0.91 | 0.84 | 0.87 | 0.91 | 0.94 | 0.91 | 0.81 | 0.86 | 0.89 | 0.95 | 0.87 | 0.82 |
| 7 | 0.84 | 0.80 | 0.93 | 0.92 | 0.84 | 0.86 | 0.90 | 0.94 | 0.91 | 0.82 | 0.86 | 0.88 | 0.95 | 0.90 | 0.84 |
| 8 | 0.81 | 0.73 | 0.92 | 0.91 | 0.82 | 0.86 | 0.90 | 0.94 | 0.91 | 0.83 | 0.86 | 0.86 | 0.94 | 0.90 | 0.84 |
| 9 | 0.80 | 0.71 | 0.93 | 0.91 | 0.80 | 0.86 | 0.88 | 0.94 | 0.91 | 0.81 | 0.84 | 0.84 | 0.94 | 0.89 | 0.82 |
| 10 | 0.81 | 0.73 | 0.92 | 0.91 | 0.79 | 0.87 | 0.89 | 0.94 | 0.91 | 0.80 | 0.86 | 0.86 | 0.94 | 0.90 | 0.82 |
| 11 | 0.82 | 0.78 | 0.93 | 0.90 | 0.78 | 0.86 | 0.90 | 0.94 | 0.90 | 0.79 | 0.84 | 0.88 | 0.94 | 0.89 | 0.79 |
| 12 | 0.86 | 0.84 | 0.93 | 0.90 | 0.80 | 0.89 | 0.92 | 0.94 | 0.90 | 0.81 | 0.86 | 0.90 | 0.94 | 0.89 | 0.81 |
| 13 | 0.89 | 0.88 | 0.94 | 0.91 | 0.83 | 0.90 | 0.92 | 0.95 | 0.92 | 0.82 | 0.88 | 0.91 | 0.95 | 0.90 | 0.82 |
| 14 | 0.89 | 0.88 | 0.94 | 0.92 | 0.84 | 0.90 | 0.92 | 0.94 | 0.92 | 0.81 | 0.88 | 0.90 | 0.94 | 0.90 | 0.81 |
| 15 | 0.91 | 0.89 | 0.94 | 0.92 | 0.86 | 0.91 | 0.93 | 0.95 | 0.92 | 0.82 | 0.90 | 0.91 | 0.95 | 0.91 | 0.83 |
| 16 | 0.92 | 0.89 | 0.94 | 0.91 | 0.84 | 0.90 | 0.92 | 0.94 | 0.91 | 0.81 | 0.89 | 0.90 | 0.94 | 0.89 | 0.81 |
| 17 | 0.91 | 0.89 | 0.94 | 0.92 | 0.85 | 0.91 | 0.92 | 0.94 | 0.92 | 0.81 | 0.89 | 0.90 | 0.94 | 0.90 | 0.82 |
| 18 | 0.93 | 0.91 | 0.94 | 0.91 | 0.84 | 0.91 | 0.93 | 0.94 | 0.92 | 0.81 | 0.90 | 0.91 | 0.94 | 0.90 | 0.81 |
| 19 | 0.93 | 0.90 | 0.93 | 0.88 | 0.82 | 0.91 | 0.92 | 0.93 | 0.90 | 0.80 | 0.89 | 0.90 | 0.93 | 0.87 | 0.80 |
| 20 | 0.93 | 0.89 | 0.92 | 0.86 | 0.82 | 0.91 | 0.92 | 0.93 | 0.89 | 0.79 | 0.89 | 0.89 | 0.93 | 0.86 | 0.79 |
|  |  | | | | |  | | | | |  | | | | |
| **SD** | **SL_110n** | | | | | **CL_20n20p** | | | | | **CL_20n2p-15&8** | | | | |
| **Phase** | **LV** | **RV** | **LA** | **RA** | **LVM** | **LV** | **RV** | **LA** | **RA** | **LVM** | **LV** | **RV** | **LA** | **RA** | **LVM** |
| 1 | 0.06 | 0.06 | 0.02 | 0.06 | 0.07 | 0.02 | 0.01 | 0.02 | 0.04 | 0.02 | 0.03 | 0.02 | 0.01 | 0.10 | 0.03 |
| 2 | 0.05 | 0.05 | 0.02 | 0.04 | 0.05 | 0.02 | 0.01 | 0.03 | 0.04 | 0.01 | 0.02 | 0.01 | 0.00 | 0.08 | 0.02 |
| 3 | 0.04 | 0.05 | 0.03 | 0.04 | 0.04 | 0.02 | 0.01 | 0.02 | 0.03 | 0.04 | 0.02 | 0.01 | 0.01 | 0.09 | 0.03 |
| 4 | 0.06 | 0.05 | 0.02 | 0.02 | 0.03 | 0.02 | 0.01 | 0.03 | 0.03 | 0.03 | 0.02 | 0.01 | 0.01 | 0.07 | 0.01 |
| 5 | 0.07 | 0.06 | 0.03 | 0.05 | 0.03 | 0.03 | 0.01 | 0.03 | 0.05 | 0.03 | 0.03 | 0.01 | 0.01 | 0.08 | 0.02 |
| 6 | 0.10 | 0.10 | 0.04 | 0.03 | 0.03 | 0.03 | 0.01 | 0.03 | 0.03 | 0.04 | 0.04 | 0.02 | 0.00 | 0.08 | 0.03 |
| 7 | 0.13 | 0.12 | 0.04 | 0.02 | 0.06 | 0.04 | 0.03 | 0.03 | 0.02 | 0.04 | 0.05 | 0.04 | 0.01 | 0.06 | 0.04 |
| 8 | 0.17 | 0.20 | 0.05 | 0.02 | 0.08 | 0.05 | 0.04 | 0.02 | 0.03 | 0.04 | 0.06 | 0.06 | 0.01 | 0.06 | 0.05 |
| 9 | 0.18 | 0.24 | 0.05 | 0.02 | 0.10 | 0.05 | 0.05 | 0.02 | 0.03 | 0.04 | 0.06 | 0.09 | 0.01 | 0.05 | 0.03 |
| 10 | 0.15 | 0.19 | 0.04 | 0.02 | 0.10 | 0.04 | 0.02 | 0.01 | 0.03 | 0.04 | 0.06 | 0.05 | 0.00 | 0.04 | 0.05 |
| 11 | 0.17 | 0.20 | 0.04 | 0.03 | 0.10 | 0.05 | 0.03 | 0.01 | 0.03 | 0.02 | 0.06 | 0.05 | 0.01 | 0.05 | 0.03 |
| 12 | 0.14 | 0.15 | 0.02 | 0.03 | 0.07 | 0.04 | 0.01 | 0.02 | 0.04 | 0.03 | 0.06 | 0.04 | 0.02 | 0.05 | 0.01 |
| 13 | 0.10 | 0.09 | 0.01 | 0.03 | 0.04 | 0.04 | 0.01 | 0.01 | 0.02 | 0.03 | 0.05 | 0.04 | 0.01 | 0.04 | 0.02 |
| 14 | 0.10 | 0.10 | 0.01 | 0.03 | 0.02 | 0.04 | 0.02 | 0.01 | 0.02 | 0.01 | 0.04 | 0.03 | 0.01 | 0.04 | 0.01 |
| 15 | 0.07 | 0.07 | 0.01 | 0.04 | 0.02 | 0.03 | 0.01 | 0.01 | 0.02 | 0.02 | 0.04 | 0.03 | 0.01 | 0.04 | 0.02 |
| 16 | 0.05 | 0.06 | 0.01 | 0.04 | 0.03 | 0.03 | 0.02 | 0.01 | 0.03 | 0.01 | 0.03 | 0.03 | 0.01 | 0.06 | 0.01 |
| 17 | 0.06 | 0.07 | 0.01 | 0.02 | 0.01 | 0.03 | 0.02 | 0.01 | 0.02 | 0.02 | 0.04 | 0.04 | 0.00 | 0.04 | 0.01 |
| 18 | 0.03 | 0.04 | 0.01 | 0.02 | 0.05 | 0.01 | 0.01 | 0.01 | 0.02 | 0.01 | 0.02 | 0.02 | 0.01 | 0.03 | 0.02 |
| 19 | 0.02 | 0.02 | 0.02 | 0.03 | 0.06 | 0.01 | 0.01 | 0.00 | 0.02 | 0.02 | 0.03 | 0.03 | 0.01 | 0.04 | 0.03 |
| 20 | 0.03 | 0.03 | 0.02 | 0.06 | 0.05 | 0.02 | 0.00 | 0.00 | 0.03 | 0.02 | 0.02 | 0.02 | 0.01 | 0.05 | 0.02 |

**Supplementary Table S4.** Patient-level Dice values for the full SL, full CL, and two-phase CL models across the four independent TL patients, corresponding to Figure 5. For each patient, the mean and standard deviation were calculated across 20 phases.

| **Mean** | **SL_110n** | | | | | **CL_20n20p** | | | | | **CL_20n2p-15&8** | | | | |
| --- | --- | --- | --- | --- | --- | --- | --- | --- | --- | --- | --- | --- | --- | --- | --- |
| **Patient** | **LV** | **RV** | **LA** | **RA** | **LVM** | **LV** | **RV** | **LA** | **RA** | **LVM** | **LV** | **RV** | **LA** | **RA** | **LVM** |
| TL1 | 0.94 | 0.92 | 0.94 | 0.90 | 0.80 | 0.92 | 0.92 | 0.94 | 0.89 | 0.83 | 0.91 | 0.91 | 0.94 | 0.88 | 0.82 |
| TL2 | 0.93 | 0.90 | 0.94 | 0.90 | 0.84 | 0.91 | 0.92 | 0.94 | 0.94 | 0.79 | 0.90 | 0.91 | 0.94 | 0.92 | 0.80 |
| TL3 | 0.90 | 0.83 | 0.95 | 0.92 | 0.86 | 0.89 | 0.90 | 0.94 | 0.92 | 0.81 | 0.88 | 0.88 | 0.94 | 0.92 | 0.82 |
| TL4 | 0.75 | 0.72 | 0.90 | 0.86 | 0.79 | 0.86 | 0.91 | 0.92 | 0.88 | 0.81 | 0.82 | 0.86 | 0.94 | 0.80 | 0.81 |
|  |  | | | | |  | | | | |  | | | | |
| **SD** | **SL_110n** | | | | | **CL_20n20p** | | | | | **CL_20n2p-15&8** | | | | |
| **Patient** | **LV** | **RV** | **LA** | **RA** | **LVM** | **LV** | **RV** | **LA** | **RA** | **LVM** | **LV** | **RV** | **LA** | **RA** | **LVM** |
| TL1 | 0.02 | 0.02 | 0.01 | 0.02 | 0.05 | 0.02 | 0.01 | 0.01 | 0.02 | 0.04 | 0.02 | 0.02 | 0.01 | 0.02 | 0.05 |
| TL2 | 0.01 | 0.02 | 0.01 | 0.05 | 0.03 | 0.02 | 0.01 | 0.01 | 0.01 | 0.02 | 0.01 | 0.02 | 0.01 | 0.03 | 0.02 |
| TL3 | 0.04 | 0.11 | 0.01 | 0.02 | 0.01 | 0.02 | 0.03 | 0.01 | 0.01 | 0.01 | 0.02 | 0.04 | 0.01 | 0.02 | 0.02 |
| TL4 | 0.12 | 0.14 | 0.03 | 0.04 | 0.08 | 0.04 | 0.03 | 0.03 | 0.02 | 0.01 | 0.04 | 0.04 | 0.01 | 0.06 | 0.01 |
|  |  | | | | |  | | | | |  | | | | |
